# Curcumin Inhibits Proliferation of Synovial Cells by Downregulating Expression of *Matrix Metalloproteinase‐3* in Osteoarthritis

**DOI:** 10.1111/os.12412

**Published:** 2018-12-17

**Authors:** Jian‐jun Zeng, Hai‐dong Wang, Zhong‐wei Shen, Xiao‐dong Yao, Cheng‐jun Wu, Tao Pan

**Affiliations:** ^1^ Department of Orthopaedics and Traumatology Jiaxing Hospital of Traditional Chinese Medicine Jiaxing China

**Keywords:** Curcumin, *MMP3*, Osteoarthritis, Synovial cells

## Abstract

**Objective:**

To investigate the association between curcumin and the differentially expressed genes (DEG) in synovial tissues of osteoarthritis.

**Methods:**

Microarray analysis was used to screen for the DEG in osteoarthritis synovial cells. Curcumin‐related genes were identified through the drug–gene interaction network STITCH (http://stitch.embl.de/cgi/input.pl). Expression levels of fibronectin 1 (FN1) and collagen III protein were measured by western blot. MTT assay was used to examine the effects of different concentrations of curcumin on cell viability. Western blot and quantitative real‐time polymerase chain reaction were used to validate the different expression levels of matrix metalloproteinase‐3 (MMP3). Clone formation assay, flow cytometry, and the TUNEL method were conducted for detecting the cell proliferation and apoptosis rate.

**Results:**

In the two chips of GSE1919 and GSE55235, the average expression of *MMP3* in the osteoarthritis group was 63.7% and 12.9% higher than that of the healthy control, respectively. The results of western blot also showed that the average expression of *MMP3* in 30 osteoarthritis patients was 132% higher than that of the healthy group, which confirmed that *MMP3* was highly expressed in osteoarthritis group. The results of MTT showed that at 72 h, the cell viability of 40 μmol/L curcumin was the lowest and 79.6% lower than for the 0 μmol/L group, so the final curcumin concentration of 40 μmol/L was selected for subsequent experiments. Western blot results further showed that the expression of *MMP3* was 44% lower in the untreated groups compared with the curcumin group, and the expressions of FN1 and collagen III were increased by 112% and 84%, respectively, which indicated that curcumin inhibited *MMP3* expression and decreased osteoarthritis synovial cell activity. Cloning formation experiments showed that cell numbers increased by 75% and 20.5% in untreated and curcumin groups, and compared with the untreated group, the cells in the curcumin group decreased by 30.8%. Flow cytometry showed that the apoptotic rate in the curcumin group increased by 85.1% compared with the untreated group, but for a single group, *MMP3* decreased the apoptotic rate by 53.9% and 46.7%, respectively.

**Conclusions:**

*MMP3* was highly expressed in osteoarthritis synovial cells. Curcumin could reduce cell viability, inhibit cell proliferation, increase cell apoptosis, and eventually alleviate inflammation of osteoarthritis by inhibiting the expression of *MMP3.*

## Introduction

Osteoarthritis and rheumatoid arthritis are the most common forms of joint inflammation. Osteoarthritis mainly affects large joints, such as buttocks, knees and finger joints^1^, causing joint pain, swelling, stiffness, osteophyte formation, dyskinesia, and functional impairment associated with activities. Osteoarthritis is often accompanied with various degrees of local inflammation, which can be diagnosed using imaging techniques and following clinical guidelines^2^. Despite the high incidence of osteoarthritis, there is currently no effective treatment to cure or reverse the progression of the disorder^3^. Surgical treatments such as osteotomy and joint replacement are viable options^4^. Steroids and non‐steroidal anti‐inflammatory drugs are the main pain‐relieving drugs^5^. However, they cannot be taken long term due to their limited efficacy and side effects, including severe cardiovascular and gastrointestinal complications^6^. Therefore, there is a need for better safety and efficacy in osteoarthritis treatment. Curcumin is characterized by its unique structure, remarkable activity, high curative effects, and minimal side effects, potentially providing a new treatment option for various diseases, including osteoarthritis^7^.

Curcumin is the main active substance and coloring component extracted from the rhizome of *Curcuma longa.* Its molecular structure contains two o‐methylated phenols and one β‐dike tone, which presents orange yellow crystalline powder with poor solubility and stability^8^. Curcumin has been widely used as a natural food pigment and wound‐healing agent, and it has also been used in the study and treatment of clinical diseases due to a variety of pharmacological properties, such as antioxidant, antimicrobial, anti‐inflammatory, and anti‐tumor properties^9^. Curcumin has been shown to significantly reduce the symptoms of osteoarthritis, to slow down the progression, and to have great potential in the treatment of osteoarthritis^10^. Studies have indicated that curcumin effectively reduced the inflammatory response of osteoarthritis by inhibiting the activation of NF‐κB and activator protein 1 (AP‐1) and the expression of inflammatory mediators, including cyclooxygenase‐2 (COX‐2), prostaglandin E2 (PGE2), nitric oxide synthase 1 (NO), interleukin 6 (IL‐6), and interleukin 8 (IL‐8)^11^, ^12^. Moreover, curcumin has been shown to have anti‐catabolic effects, potentially by inhibiting matrix metalloproteinase (MMP), which could catalyze the decomposition of cartilage extracellular matrix^13^, prevent cartilage degradation, and promote the accumulation of newly synthesized matrix components in the extracellular matrix. In addition, it has been suggested that curcumin could also enhance the cell survival rate and resist the cytotoxicity induced by Interleukin 1 beta (IL‐1β)^3^.

Matrix metalloproteinase is a zinc‐dependent protease that not only has a crucial role in extracellular decomposition processes but also has an extracellular matrix (ECM) in pathology such as arthritis and tumor invasion^14^. *MMP3* is a member of the MMP family and a joint modulator of ECM involved in disease morphogenesis, wound healing, tissue repair, and remodeling^15^. Ma *et al*. showed that the expression levels of serum *MMP3* and inflammatory mediators in rheumatoid arthritis were positively correlated with the severity of the disease. Therefore, *MMP3* can potentially be used to diagnose rheumatoid arthritis with non‐invasive biomarkers^16^. In addition, Chen *et al*. found that lycorine can significantly reduce IL‐1β, and induce MMP‐3 and MMP‐13 mRNA and protein expression, which can protect the cartilage and treat osteoarthritis^17^. Furthermore, Yang *et al*. found that NAMPT exerts its catabolic function by downregulating aggrecan and upregulating *MMP3* and *MMP13*, which is a key characteristic of osteoarthritis pathogenesis and a direct target of Hypoxia‐inducible factor 2‐alpha (HIF‐2α) in articular chondrocytes and osteoarthritis cartilage^18^. In this study, we mainly investigated the effects of *MMP3* on synovial cells of osteoarthritis, and explored the regulating effects of curcumin on the expression of *MMP3.*

In summary, we validated the expression of *MMP3* in osteoarthritis by western blot and quantitative real‐time polymerase chain reaction (qRT‐PCR), and examined the inhibitory effect of curcumin on *MMP3.* Experiments on cellular functions revealed that curcumin inhibited the proliferation of osteoarthritis synovial cells, promoted apoptosis, and relieved osteoarthritis inflammation by inhibiting the expression of *MMP3.*

## Materials and Methods

### *Bioinformatics Analysis*

GSE1919 (GPL91), which contained 5 tissue samples of patients with osteoarthritis and 5 healthy controls, and GSE55235 (GPL96), which contained 10 tissue samples of patients with osteoarthritis and 10 healthy controls, were selected for targeted chip research from the GEO database. Differentially expressed genes were screened using the “limma” package, in which the screening criteria were set to be |log_2_ (Fold Change) |>1 and adj.P.val < 0.05. Then, we used the drug–gene interaction network STITCH to find curcumin‐related genes. Finally, the genes were determined by Venn analysis.

### *Collection of Clinical Samples*

With the consent of the patients, synovial samples of knee joints of 30 osteoarthritis patients and 15 healthy controls were collected from the Jiaxing Hospital of Traditional Chinese Medicine between June 2016 and June 2017. This study was performed with the approval of the Ethical Committee of Jiaxing Hospital of Traditional Chinese Medicine. All synovial specimens were collected prior to determination of adjuvant or palliative systemic treatment.

### *Cell Culture*

Synovial cells in osteoarthritis patients and healthy controls were selected. In the sterile environment, the synovial tissues collected from the clinical samples were taken out. After rinsing, the synovial tissues were cut as far as possible in the dish and placed in a sterile vial. Then 0.25% Collagenase II was added to continuously digest three times for 20 min each time. After merging the digestive juice, the liquid was sieved using a No. 6 metallic sieve. The cell suspension was placed in a centrifuge tube and centrifuged at 1110 g for 10 min; then the medium was discarded. Cells were cultured in Dulbecco's Modified Eagle Medium containing 10% fetal bovine serum (FBS) and inoculated into cell culture bottles at a density of 1 × 10^5^ cells/mL. Subsequently, cells were incubated at 37°C with 5% CO_2_ and saturated humidity. The culture medium was replaced every other day. When the cells covered 80% of the culture bottle, the cells were digested and subcultured with 0.25% trypsin.

### *Cell Transfection*

pcDNA3.1‐*MMP3*, si‐*MMP3*, and negative control (NC) sequences were purchased from Shanghai Genechem (Shanghai, China). The sequences of si‐*MMP3* and NC are shown in Table 1. Interfering nucleotide sequence was designed according to Invitrogen RNA interference sequence design software (BLOCK‐iTTM RNAi Designer). At the same time, a negative control was designed according to an unrelated nucleic acid sequence, which has the same base number. Cells were transformed until the competent cells amplified; positive clones were picked, and the recombinant plasmid was obtained and identified for sequence. Cells were seeded 1 day before the transfection and cells in the logarithmic growth phase were cultured in serum antibiotic‐free medium at 37°C with 5% CO_2_. Cells in good condition were placed on 6‐well plates. When the cell density reached 80%, a transfection experiment was conducted, with transfection mixture prepared according to the instructions for the Lipofectamine 2000 (Invitrogen; Thermo Fisher Scientific, Waltham, MA, USA) kit. The media solution was discarded, the prepared transfection mixture was added to the plates, and the cells were incubated at 37°C. Cells were divided into three groups: an NC group (osteoarthritis synovial cells transfected with negative control); an si‐*MMP3* group (osteoarthritis synovial cells transfected with si‐*MMP3*); and an *MMP3* group (transfected with *MMP3* of osteoarthritis synoviocytes).

**Table 1:** Sequences of si‐RNA

Genes	Sequences (5′‐3′)
Si‐MMP3	CCATTGGATGGAGCTGCAA
NC	CCATAGGGAGGGTCTTCAA

MMP3, matrix metalloproteinase‐3; NC, negative control.

### *Western Blot*

The cells in the logarithmic growth phase were selected and washed with phosphate buffer saline (PBS), and the lysate was added. The total proteins were extracted by centrifugation to remove impurities. Protein concentrations were determined by BCA assay (Solarbio, China) and for each sample, 50 μg were resolved on 4%–15% Trise HCl SDS‐polyacrylamide gels, and blotted onto immobilon polyvinylidene fluoride for immunoblotting. The blots were incubated with 5% non‐fat dry milk in tris buffered saline tween for 1 h at room temperature and then stained with anti‐*MMP3* (ab52915, 1:500; Abcam, Cambridge, MA, USA), anti‐collagen III (ab7778, 1:5000), anti‐FN1 (ab23750, at a concentration of 1 μg/mL), and anti‐GAPDH (ab9485, 1:500) at 4°C overnight. After incubation with primary antibodies, membranes were washed, incubated with horseradish secondary antibody IgG (ab205718, 1:5000) for 1 h, and washed again. Protein band densities were quantified using an enhanced chemiluminescence detection system (syngene, USA). The protein levels were analyzed using the Image J software.

### *MTT Assay*

The cells were pretreated with 5, 10, 20, and 40 μmol/L curcumin solution separately when the cells were adhered completely. After adding 10 μL MTT solution (5 mg/mL) separately, the cells were cultured at 37°C for 4 h. Then the medium was carefully aspirated, and 150 μL dimethyl sulfoxide was added. Finally, the optical density (OD) value of each group was determined using an ultraviolet spectrophotometer with 490 nm as the reference wavelength.

### *Colony Formation*

In the presence of 10% human serum or medium containing 10% FBS, cells were cultured in two groups: untreated and curcumin‐treated groups. Then *MMP3* was knocked down and overexpressed in both groups, respectively. The media were changed every 3 days. Subsequently, the cells were fixed with cold methanol for 5 min and washed with PBS before being incubated with crystal violet (Sigma, USA) for 30 min. Then the excess stains were rinsed with tap water. The cells were air‐dried and then observed under a microscope.

### *Cell Apoptosis Analysis*

The cell apoptosis rate was detected by flow cytometry. Cells transfected for 24 h were digested by trypsin, centrifuged, and collected. The cells obtained were washed with cold PBS, then made into 1 × 10^6^ cells/mL single cell suspension with 500 μL binding buffer solution (calcium containing PBS). At room temperature, 100 μL of cell suspension was added into the tube, followed by 5 μL Annexin V‐FITC (BD Biosciences, Franklin Lakes, NJ, USA) and 5 μL propidium iodide (BD Biosciences). After 30 min of incubation at 4°C, 400 μL binding buffer was added immediately before the detection by the flow cytometry (BD Biosciences), with 1 × 10^4^ cells each time. The data was analyzed using Cell Quest (BD Biosciences) to calculate the rate of cell apoptosis.

### *TUNEL Assay*

DNA fragmentation was measured using a one‐step TUNEL Apoptosis Assay Kit (Roche, Germany) according to the kit's manual. The images were captured with a Nikon ECLIPSE Ti Microscope (Nikon, Japan).

### *Quantitative Real‐time Polymerase Chain Reaction Assays*

Total RNA was isolated using TRIzol Reagent (Invitrogen) and quantified by the PrimeScript 1st Strand Synthesis Kit (TaKaRa, Tokyo, Japan); 2 μL cDNA was performed using the QuantiTect SYBR® Green RT‐PCR Kit (QIAGEN, Duesseldorf, Germany) on the ABI 7500 Real‐time PCR System (Applied Biosystems). Primers used are shown in Table 2. Samples were analyzed in duplicate, using housekeeping GAPDH as an endogenous control.

**Table 2:** Primer sequences for quantitative real‐time polymerase chain reaction

Genes	Sequences
MMP3	F: 5′‐CCCGAGGTTGGACCTACAAG ‐3′
	R: 5′‐CTTCCCCGTCACCTCCAATC ‐3′
GAPDH	F: 5′‐AGTAGAGGCAGGGATGATG ‐3′
	R: 5′‐TGGTATCGTGGAAGGACTC ‐3′

MMP3, matrix metalloproteinase‐3.

### *Statistical Analysis*

The data was presented as mean ± standard deviation (SD) from three separate experiments. Differences among groups were analyzed by Student's *t*‐test or one‐way ANOVA using Graphpad Prism 6 software (La Jolla, CA, USA). Refutation of the null hypothesis was accepted when *P*‐values were less than 0.05.

## Results

### *Matrix Metalloproteinase‐3 Was Highly Expressed in Osteoarthritis*

**Figure 1:**
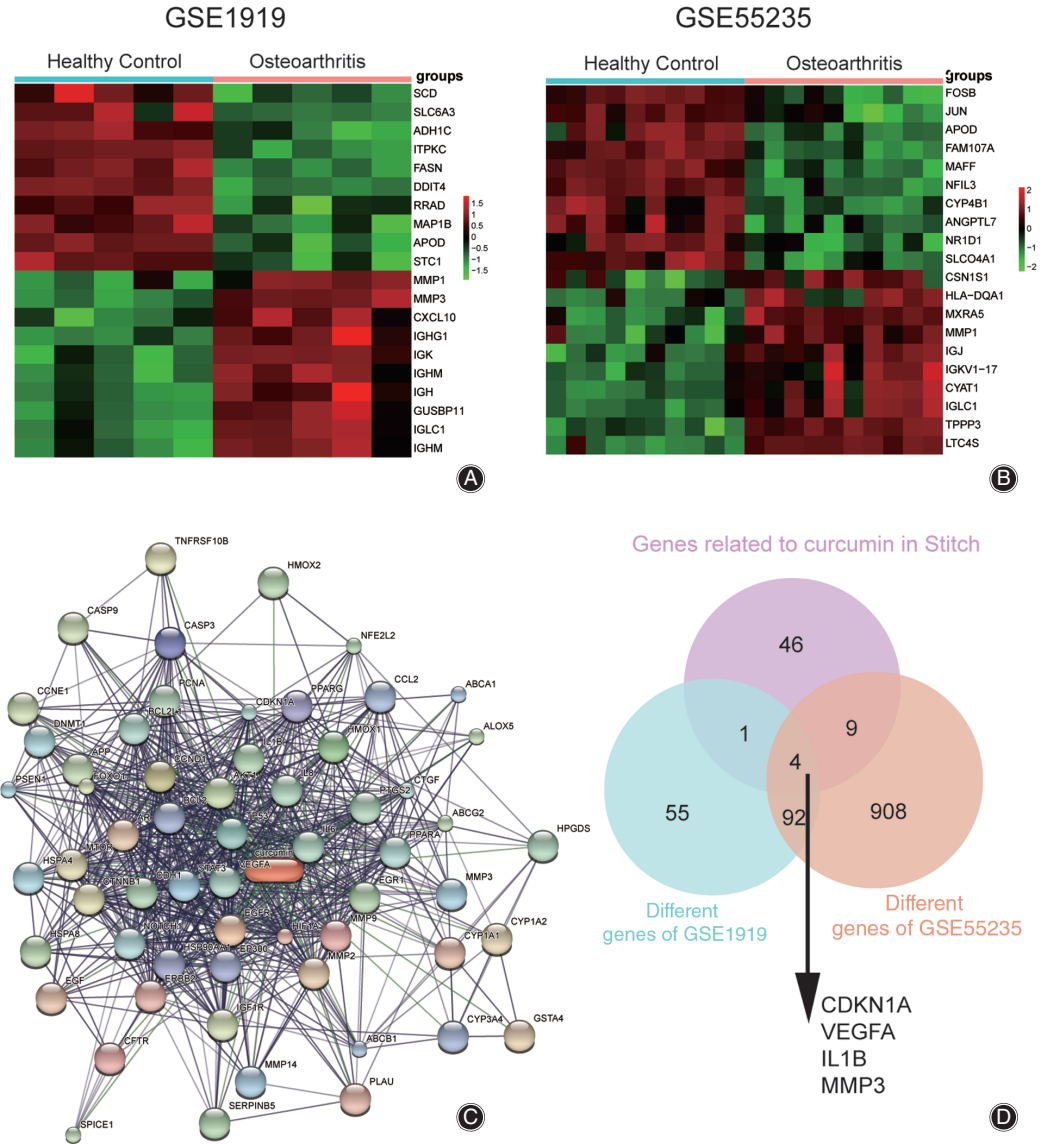
Chip analysis and gene screening. (A) Unsupervised top 20 differential gene analysis with GSE1919, based on the limitation of |log_2_(Fold Change)|>1 and adj.*P.*val < 0.05. (B) Unsupervised top 20 differential gene analysis with GSE55235, based on the limitation of |log_2_(Fold Change)|>1 and adj.*P.*val < 0.05. (C) The genes related to curcumin were found in STITCH. (D) Four overlapping genes were found by Venn analysis.

To determine the differentially expressed genes in osteoarthritis patients, an integrated database of two microarrays was analyzed. GSE1919 and GSE55235 were included in the analysis. Microarray analysis presented the results of the first 20 differentially expressed genes under the conditions of |log_2_(Fold Change) |>1 and adj.P.val < 0.05 (Fig. 1A, B). Meanwhile, we identified genes related to curcumin through STITCH (Fig. 1C). We then compared the differentially expressed genes derived from GSE1919 and GSE55235. Four genes both differentially expressed and related to curcumin were found in all three locations (Fig. 1D). After careful review of the recent literature, results for vascular endothelial growth factor A (VEGFA) and interleukin 1 beta (IL1β) were diametrically opposite to those in our osteoarthritis microarray analysis, so we excluded them. Meanwhile, we found that cyclin‐dependent kinase inhibitor 1A (CDKN1A) was less studied in osteoarthritis. Finally, we selected *MMP3* for the follow‐up studies.

**Figure 2:**
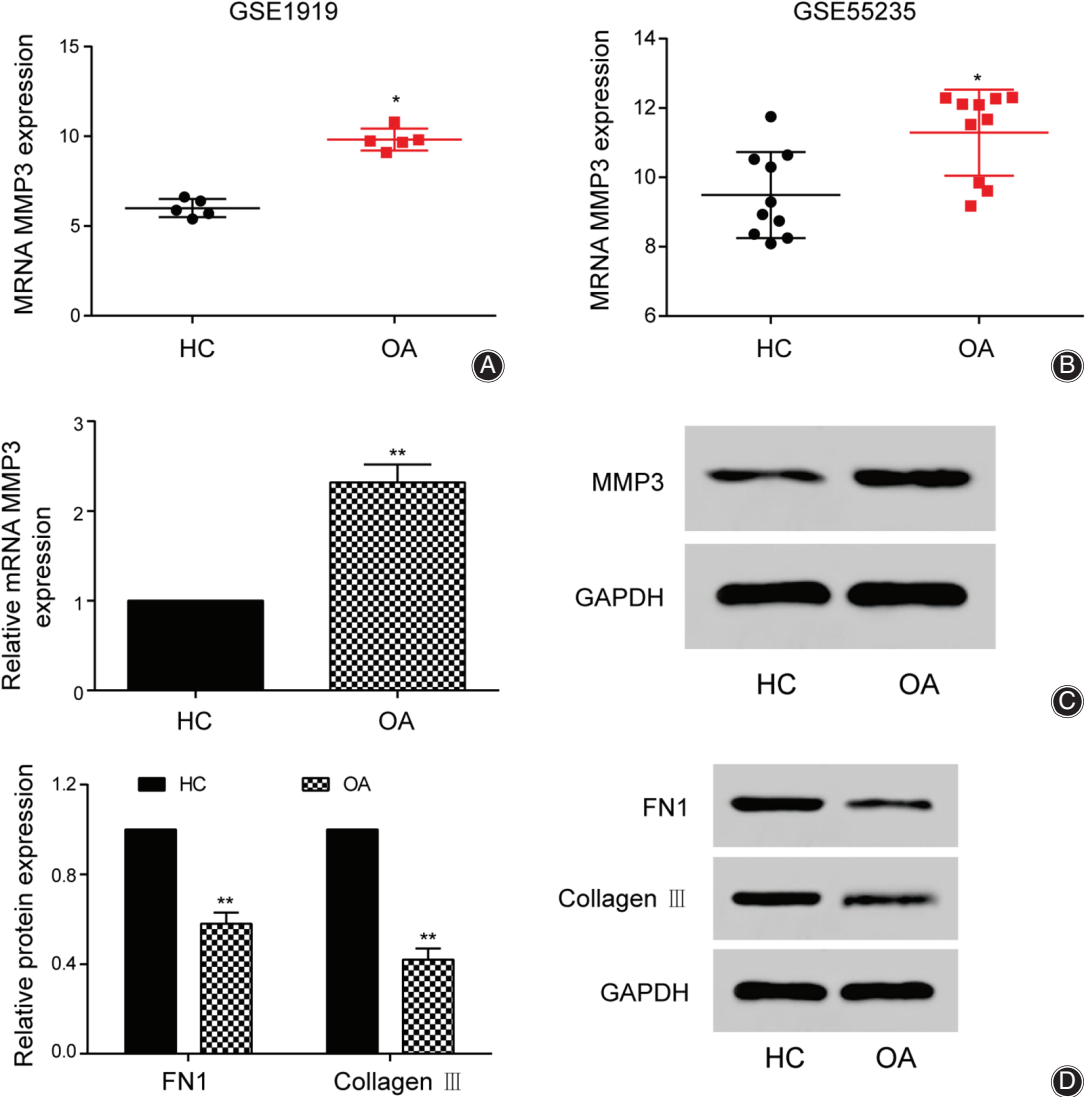
*MMP3* was highly expressed in osteoarthritis. (A) The expression of *MMP3* in healthy control group and Osteoarthritis group were performed according to GSE1919. (B) The expression of *MMP3* in healthy control group and Osteoarthritis group were performed according to GSE55235. (C) The relative expression levels of *MMP3* were detected in the healthy control group and the osteoarthritis group by quantitative real‐time polymerase chain reaction (qRT‐PCR) and western blot. (D) The different expression of FN1 and collagen III protein in healthy control and Osteoarthritis group by qRT‐PCR and western blot. *P < 0.05 and **P < 0.01 indicated statistical significance compared with healthy control group.

Reanalyzing the expression of *MMP3* in GSE1919 (*n* = 5) and GSE55235 (*n* = 10) showed that compared with normal synovial cells (healthy control group), *MMP3* was highly expressed in the synovial cells of osteoarthritis (Fig. 2A, B, *P* < 0.05). In addition, the relative expression levels of MMP3 detected in synovial cells of osteoarthritis patients and normal donors by western blot and qRT‐PCR were consistent with microarray results. The western blot results also showed that the average expression of MMP3 in synovial cells of osteoarthritis was 132% higher than that of the healthy group (Fig. 2C, *P* < 0.01). The expression of the two downstream target genes of MMP3 identified, FN1 and collagen III, were also examined. Based on previous studies on osteoarthritis, we found that these two genes were closely related to the occurrence and development of osteoarthritis^19^, ^20^. Results from both qRT‐PCR and western blot showed that in the osteoarthritis group, FN1 and collagen III expression levels were, respectively, 42% and 58% lower than in the healthy control group (Fig. 2D, *P* < 0.01).

### *Curcumin Inhibited Matrix Metalloproteinase‐3 Expression and Reduced the Activity of Synovial Cells in Osteoarthritis*

**Figure 3:**
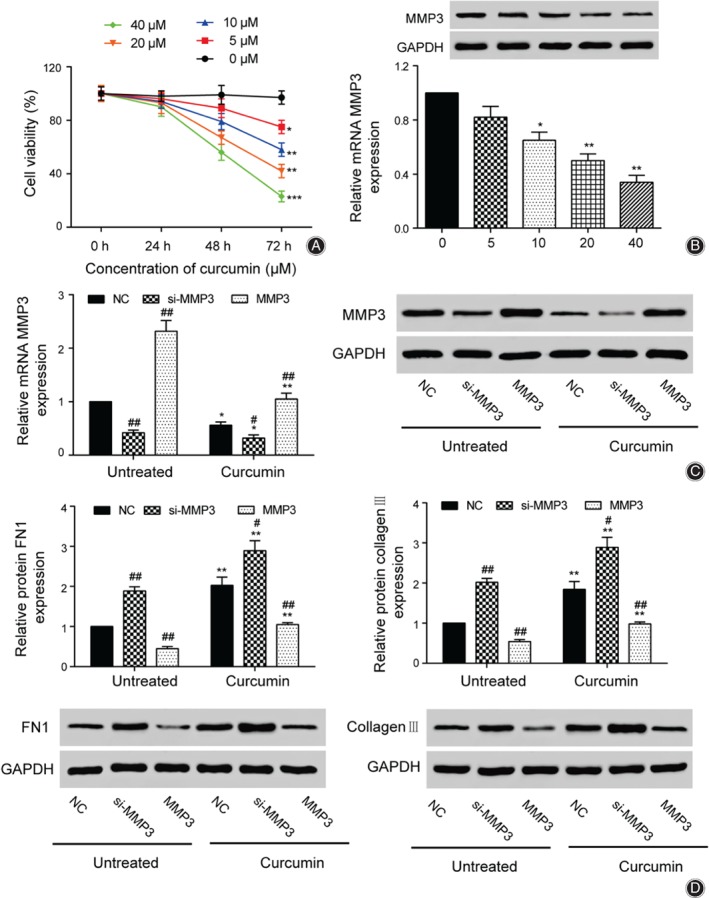
Curcumin inhibited *MMP3* expression and reduced the viability of synovial cells in osteoarthritis. (A) MTT was used to measure the cell viability under different concentrations of curcumin. (B) Western blot and quantitative real‐time polymerase chain reaction (qRT‐PCR) were used to detect the different levels of curcumin expression. (C) The expression levels of *MMP3* were detected in the untreated group and the curcumin group by qRT‐PCR and western blot. (D) The expression levels of FN1 and collagen III in the untreated group and the curcumin group were detected by western blot. ^**#**^ *P* < 0.05 and ^**##**^ *P* < 0.01 suggested statistical significance compared with the negative control (NC) group, respectively. *P < 0.05 and **P < 0.01 suggested statistically significant difference compared to the corresponding group in the untreated group.

The cell viability of synovial cells was examined at different concentrations of curcumin (0, 5, 10, 20, and 40 μmol/L). Cell viability was lower in the curcumin‐treated groups than in the untreated group, and decreased to the lowest level after 72 h in the curcumin‐treated groups. Furthermore, the cell viability of 40 μmol/L curcumin was the lowest and 79.6% lower than in the 0 μmol/L group. The results showed that cell viability varied with the concentration of curcumin and the exposure time of treatment (Fig. 3A). Western blot and qRT‐PCR were then used to detect the levels of MMP3. MMP3 expression decreased as the curcumin concentration increased. At 40 μmol/L curcumin, the expression level of MMP3 was at its lowest and 66% lower than for the 0 μmol/L group, which was also observed in the western blot results (Fig. 3B). We then knocked down and overexpressed *MMP3*, respectively, and performed western blot and qRT‐PCR assays for both treatment and non‐treatment groups. The expression levels of MMP3 in the curcumin group were 44% lower in the treated groups than the untreated groups. Moreover, the MMP3 level was 68% lower in the si‐*MMP3* group than the NC group in the untreated and curcumin‐treated groups, and was 132% higher in the overexpression group. The same results were found for western blot (Fig. 3C). These results implied that curcumin could inhibit the expression of MMP3. We also measured FN1 and collagen III protein levels in each group. For FN1, the expression of NC, si‐*MMP3*, and *MMP3* in the curcumin group increased by 112%, 68%, and 70.1% compared with the untreated group, respectively. For collagen III, the expression of NC, si*‐MMP3*, and *MMP3* in the curcumin group increased by 84%, 41.1%, and 41.3% compared with the untreated group, respectively (Fig. 3D).

### *Matrix Metalloproteinase‐3 Could Promote the Proliferation of Synovial Cells and Inhibit Apoptosis*

**Figure 4:**
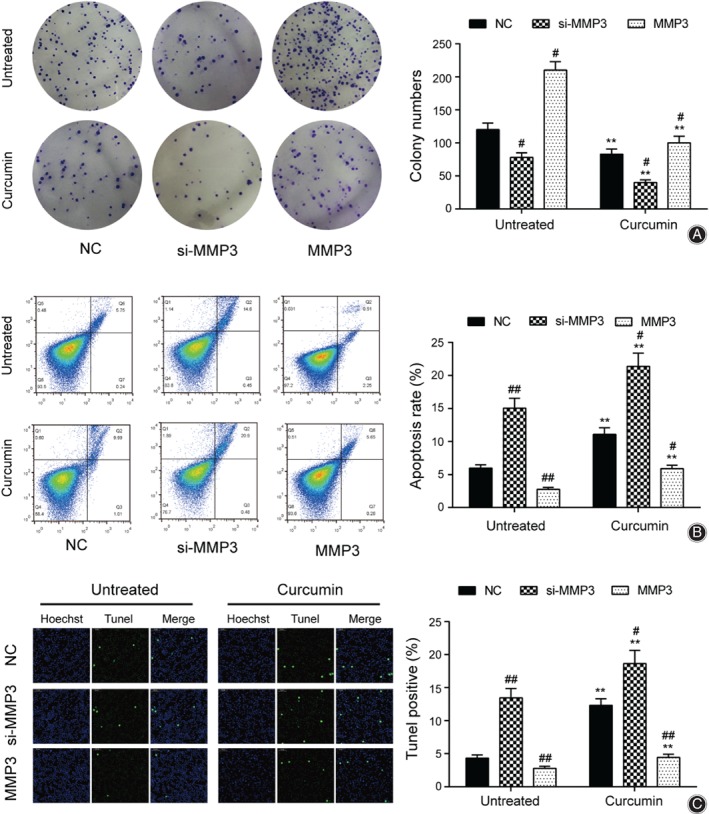
*MMP3* could promote the proliferation of synovial cells and inhibit apoptosis. (A) Cell proliferation in untreated group and curcumin group was measured by colony formation assay. (B) Apoptosis in untreated group and curcumin group was tested by cell apoptosis. (C) Apoptosis in untreated group and curcumin group was evaluated by TUNEL method. ^**#**^ *P* < 0.05 and ^**##**^ *P* < 0.01 suggested statistical significance compared with the negative control (NC) group, respectively. *P < 0.05 and **P < 0.01 suggested statistically significant difference compared to the corresponding group in the untreated group.

The functional properties of synovial cells with altered levels of *MMP3* were tested. The clone formation experiment showed that the numbers of clones in the curcumin group decreased 30.8%, 48.7%, and 52.4% compared with the untreated in all NC, *MMP3* knockdown, and overexpression groups. In addition, the si‐*MMP3*‐treated groups showed significantly fewer colonies than the NC groups, and the *MMP3*‐overexpressing groups showed significantly more colonies than the NC groups (Fig. 4A). Following the colony formation experiments, flow cytometry and TUNEL experiments were performed to detect the apoptosis rate. Compared with the untreated group, the apoptotic rates in the curcumin group increased significantly. Furthermore, apoptotic rates were increased 151.2% and 87.6% in untreated and curcumin groups with knockdown of *MMP3.* In *MMP3*‐overexpressing cells, both untreated and curcumin‐treated, the apoptosis rates were 53.9% and 46.7% lower than in the NC groups (Fig. 4B). Similar patterns were observed in the results of TUNEL experiments; the TUNEL positive of NC, si‐*MMP3*, and *MMP3* in the curcumin group increased by 185.2%, 36.8%, and 58.8% compared with the untreated group, respectively (Fig. 4C).

## Discussion

In this study, we predicted the differentially expressed gene *MMP3* by means of chip analysis, and experimentally verified its high expression in osteoarthritis synovial cells through western blot and qRT‐PCR experiments. Meanwhile, we tested the expression of FN1 and collagen III, which were all related with osteoarthritis. We also found that curcumin could inhibit the expression of *MMP3.* At the same time, the experiments of cell proliferation and cell apoptosis indicated that curcumin could promote the apoptosis of osteoarthritis by inhibiting *MMP3*, which could reduce the inflammation of osteoarthritis.

### *Matrix Metalloproteinase‐3 Plays an Important Role in Bone‐related Diseases*

Matrix metalloproteinase‐3 has been shown to be involved in a variety of inflammatory and oncological conditions. Lu *et al*. found that sodium alginate extract inhibited gene expression of pro‐inflammatory MMP such as *MMP3* in a dose‐dependent manner and increased the expression level of major aggrecan mRNA at 1000 μg/mL in articular cartilage^21^. Takamatsu *et al*. show that verapamil can inhibit Wnt‐reactive *AXIN2* and *MMP3* gene expression in human osteoarthritis chondrocytes, and, thus, relieve symptoms of osteoarthritis effectively^22^. Wang *et al*. found that *MMP3* expression was significantly increased after transfection of HS6ST2‐specific interfering RNA (siRNA) into C28/12 cells, indicating that HS6ST2 may be involved in the pathogenesis of osteoarthritis and Kashin–Beck disease by affecting aggrecan metabolism^23^. Jessberger *et al*. found that Pycnogenol can reduce the expression of *MMP3*, *MMP13*, and the pro‐inflammatory cytokine IL1β, so it has some effect on severe bone and joint inflammation^24^. In our study, *MMP3* was found to be overexpressed in osteoarthritis synovial cells. Importantly, we verified that high expression of *MMP3* would promote cell proliferation and inhibit cell apoptosis, increasing osteoarthritis inflammation in the end.

### *Curcumin's Therapeutic Effect on Osteoarthritis*

Curcumin has anti‐inflammatory and anti‐oxidant functions and regulates various biochemical and molecular pathways by regulating multiple molecular targets^25^. Horcajada *et al*. demonstrate that oleuropein and rutin ± curcumin significantly delayed the progression of spontaneous osteoarthritis in guinea pigs^26^. Mobasheri *et al*. suggest that curcumin could reach prophylactic properties by inhibiting NF‐κB signaling, thus preventing inflammation in osteoarthritis, and indicating that curcumin and resveratrol could be a candidate for adjuvant treatment with nonsteroidal anti‐inflammatory drugs^27^. Wang *et al*. demonstrate that curcumin can treat osteoarthritis by inhibiting NF‐κB signaling^19^. Clutterbuck *et al*. found that non‐cytotoxic concentrations of curcumin exerted anti‐catabolic and anti‐inflammatory effects in cartilage explants and had a therapeutic effect on osteoarthritis^28^. Similarly, our study revealed that curcumin could decrease the expression of *MMP3* on osteoarthritis synovial cells, inhibit proliferation, and promote apoptosis.

### *Relationship between FN1 and Collagen III and Osteoarthritis*

FN1 and Collagen III are known as osteoarthritis regulators^20^, ^29^, and they are markers in the treatment of osteoarthritis. Chang *et al*. demonstrate that FN1 may be differentially expressed in post‐traumatic osteoarthritis compared with normal joints^20^. In a mouse model of the medial meniscus, Fn1 was a hub in a reconstructed network in osteoarthritis with the transcriptional analysis^30^. Ibuprofen upgraded the expression of types I, II, and III collagen and increased the synthesis of collagen in a rat osteoarthritis model^29^; we also tested the expression of FN1 and Collagen III under the treatment of curcumin, representing the inflammatory response of osteoarthritis. Similarly, our results demonstrated that curcumin could treat osteoarthritis through reducing the inflammatory response.

The present study has some limitations. For instance, curcumin did not only alter the expression of *MMP3* but also had effects on other proteins. *In vivo* experiments are needed to further prove our findings in a mouse model.

In conclusion, *MMP3* was overexpressed in osteoarthritic synovial cells and synovial tissue. Curcumin could downregulate the expression of *MMP3*, promote apoptosis, and inhibit cell proliferation, as well as ultimately alleviate osteoarthritis inflammatory response. This study not only provided a treatment strategy for osteoarthritis but also proved that *MMP3* may be a new biomarker for judging the prognosis of osteoarthritis.
